# Molecular Basis of Mitochondrial and Peroxisomal Division Machineries

**DOI:** 10.3390/ijms21155452

**Published:** 2020-07-30

**Authors:** Yuuta Imoto, Kie Itoh, Yukio Fujiki

**Affiliations:** 1 Department of Cell Biology, Johns Hopkins University School of Medicine, 725 N. Wolfe Street, Baltimore, MD 21205, USA; kito5@jhmi.edu; 2 Division of Organelle Homeostasis, Medical Institute of Bioregulation, Kyushu University, 3-1-1 Maidashi, Higashi-ku, Fukuoka 812-8582, Japan; 3 Institute of Rheological Functions of Food, Hisayama-cho, Fukuoka 811-2501, Japan

**Keywords:** mitochondrial division, peroxisomal division, dynamin-related protein Dnm1/Drp1, nucleoside-diphosphate kinase, local GTP generation

## Abstract

Mitochondria and peroxisomes are ubiquitous subcellular organelles that are highly dynamic and possess a high degree of plasticity. These organelles proliferate through division of pre-existing organelles. Studies on yeast, mammalian cells, and unicellular algae have led to a surprising finding that mitochondria and peroxisomes share the components of their division machineries. At the heart of the mitochondrial and peroxisomal division machineries is a GTPase dynamin-like protein, Dnm1/Drp1, which forms a contractile ring around the neck of the dividing organelles. During division, Dnm1/Drp1 functions as a motor protein and constricts the membrane. This mechanochemical work is achieved by utilizing energy from GTP hydrolysis. Over the last two decades, studies have focused on the structure and assembly of Dnm1/Drp1 molecules around the neck. However, the regulation of GTP during the division of mitochondrion and peroxisome is not well understood. Here, we review the current understanding of Dnm1/Drp1-mediated divisions of mitochondria and peroxisomes, exploring the mechanisms of GTP regulation during the Dnm1/Drp1 function, and provide new perspectives on their potential contribution to mitochondrial and peroxisomal biogenesis.

## 1. Introduction

Mitochondria and peroxisomes play vital roles in cellular metabolism. Double-membrane-bounded mitochondria contain their own DNA derived from an endosymbiotic ancestor and are the powerhouse of eukaryotic cells, playing roles in the regulation of the cellular redox state, Ca^2+^ homeostasis, and apoptosis [1,2,3]. Single-membrane-bounded peroxisomes were originally defined as a carrier of flavin-oxidases, producing H_2_O_2_ and catalase [4]. Subsequently, peroxisomes were found to exert important metabolic functions in lipid homeostasis [5]. Mitochondria and peroxisomes are metabolically linked organelles and share matrix enzyme activities, including fatty-acid β-oxidation enzymes [6]. Since mitochondria and peroxisomes share functions, their morphogenesis and number need to be coordinated. Importantly, the number of both organelles is maintained by the division of pre-existing organelles using the same division machinery [7,8]. A range of pathological conditions, including cancer, aging, neurodegeneration and metabolic diseases, are associated with disorders in mitochondrial and peroxisomal division [9,10,11]. Thereby, the divisions of mitochondria and peroxisomes have been studied extensively over the last two decades with various model systems including yeast, mammals, and algae. There are two important findings in this field. (1) The identification of dynamin-like protein Dnm1/Drp1 (Dnm1 in yeast and algae; Drp1, DRP1, or DLP1 in mammals), which catalyzes the membrane fission of mitochondria [12,13,14] and peroxisomes [7] in yeast and mammalian cells as a model system. Dnm1/Drp1 is a dynamin family member as well as a classical dynamin involved in the endocytosis [15]. Similar to classical dynamin, Dnm1/Drp1 contains a GTPase domain (G-domain) and forms a helical polymer to constrict the membrane tubules upon binding to and hydrolyzing GTP [16,17]. (2) The identification of an electron-dense “ring” around the neck of the dividing organelles. This electron-dense ring was first identified around the neck of dividing mitochondrion in a unicellular alga, *Cyanidioschyzon merolae* [18,19]. It was also observed later around the neck of a dividing peroxisome [20]. A similar electron-dense neck has also been observed around the constriction site of mitochondrion in yeast [16] and mammalian cells [21]. These findings raise a view that mitochondria and peroxisomes divide via the constriction of the ring-shaped division machinery composed of Dnm1/Drp1 [22,23]. Research in this field has previously focused on the receptor-mediated recruitment of the Dnm1/Drp1-based division machinery to membrane fission sites [24]. In addition to exploring of the function of Dnm1/Drp1 receptors, a recent study on *C. merolae* demonstrated that the energy source of Dnm1/Drp1, GTP is spatio-temporally regulated during the divisions of mitochondrion and peroxisome, raising an idea that the energy source for the organelle division machinery is locally generated [25]. In this review, we briefly compile the current knowledge about membrane remodeling of mitochondria and peroxisomes in yeast, algae, and mammalian cells. We also address molecular mechanisms underlying the energetic regulation of Dnm1/Drp1-based machineries, in regards to mitochondrial and peroxisomal division.

## 2. Mitochondrial Dynamics

### 2.1. In Yeast and Mammals

**Figure 1:**
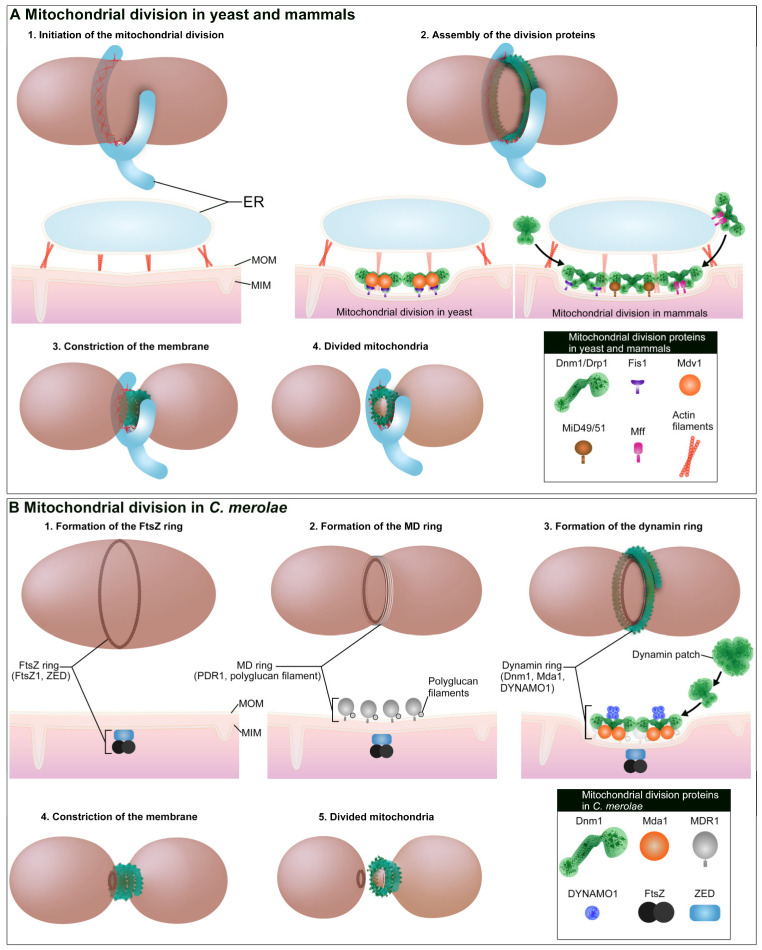
Mitochondria are divided by Dnm1/Drp1-based membrane fission machinery. Schematic images represent dividing mitochondria and their cross sections at the division plane. (**A**), Mitochondrial division in yeast and mammals is initiated at the endoplasmic reticulum (ER)-mitochondrion contact site. At the contact site, ER tubules encircle the mitochondria and polymerized actin is formed. Receptor proteins Fis1 and Mdv1/Caf4 are involved in the recruitment of Dnm1 to mitochondrial outer membrane (MOM) in yeast, while Fis1, Mff, and MiD49/51 are involved in the recruitment of Drp1 in mammals. In mammals, the ER–mitochondrion contact site also participates in the recruitment of Drp1. Polymerized Dnm1/Drp1 constricts and pinches off mitochondrial division site. (**B**) In *C. merolae*, a mitochondrion is divided by mitochondrion-dividing (MD) machinery (FtsZ ring, MD ring and dynamin ring). The first event of the mitochondrial division is formation of the FtsZ ring. ZED is important for the FtsZ ring formation on the matrix side of MIM followed by the formation of MD ring. MDR1 is involved in the formation of MD ring. Dynamin ring is composed of Dnm1, and Mdv1/Caf4 ortholog Mda1 is involved in the recruitment of Dnm1. Dnm1 is likely recruited from cytosolic dynamin patches. During the constriction of MD machinery, DYNAMO1 generates GTP and supports the GTPase activity of Dnm1.

In yeast and mammalian cells, membrane fission and fusion are responsible for maintaining the morphology and number of mitochondria [26,27,28]. The core component of the mitochondrial fission machinery is Dnm1/Drp1 that was first identified as Dnm1 by yeast mutagenesis [12,13,14]. Dnm1 is a dynamin-related GTPase, which is recruited to the mitochondrial outer membrane (MOM), where it self-assembles via GTP binding followed by membrane constriction (Figure 1A). Loss of Dnm1 results in long interconnected mitochondrial networks [13,14]. Receptors with transmembrane domains are involved in recruitment of Dnm1 to the MOM. Genetics studies in yeast identified the receptor proteins of Dnm1, namely mitochondrial fission 1 protein (Fis1) [29], mitochondrial division protein 1 (Mdv1) [30], and CCR4-associated factor 4 (Caf4) [31]. In mammals, a Dnm1 ortholog, Drp1 [32], and its receptor protein, Fis1, have been identified [21]. However, it has been reported that Fis1 is dispensable for Drp1-mediated mitochondrial fission in mammalian cells [33,34] and important under a specific physiological condition, such as stress-induced mitophagy [35]. More recent studies have shown that Fis1 negatively regulates mitochondrial fusion [36]. As such, Fis1 may exert functions other than in the recruitment of Drp1 in mammalian cells. Mdv1 and Caf4 are not conserved in mammals [37]. Instead, recruitment of Drp1 likely depends on other receptors, such as mitochondrial fission factor (Mff) [33], which was originally identified by the small interfering RNAscreening of the cultured *Drosophila melanogaster* cells [38], and mitochondrial dynamics proteins of 49 kDa (MiD49) and 51 kDa/mitochondrial elongation factor 1 (MiD51/MIEF1) conserved in vertebrates [39,40]. In addition, the mitochondrial distribution and morphology protein 36 (Mdm36), and a cortical protein, nuclear migration protein 1 (Num1), are also known to regulate mitochondrial fission and distribution in yeast [41,42]. Mammalian orthologs of Mdm36 and Num1 have not yet been identified.

Mitochondrial fusion is also regulated by dynamin-related proteins. The first identified mitochondrial fusion gene is *fuzzy onions* (*fzo1*) in *D. melanogaster* [43]. Fzo1 is a transmembrane protein with its GTPase domain that is exposed to the cytoplasm. A molecular genetic study using yeast found that *fzo1* mutation causes a fragmented mitochondrial phenotype as a result of blocking the fusion of MOM [44]. *Fzo1* and *Dnm1* double-mutations alter this fragmented phenotype to wild-type mitochondrial morphology, indicating that the balance between fission and fusion plays an important role in the morphology of mitochondria [14]. In yeast, the fusion of mitochondrial inner membrane (MIM) is regulated by a dynamin-related GTPase, Mgm1 [45]. In mammalian cells, the fusion of MOM is regulated by Fzo1 orthologs, namely mitofusins 1 and 2 (Mfn1 and Mfn2) [46], and MIM is regulated by the Mgm1 ortholog optic atrophy-1 (OPA-1) [47,48].

In the initiation of mitochondrial dynamics, the endoplasmic reticulum (ER) plays an important role. During mitochondrial division, ER tubules encircle and constrict mitochondrial tubules prior to the recruitment of Dnm1/Drp1 to the mitochondria [49]. At the ER-marked mitochondrial division site, an ER-associated formin, INF2, facilitates the polymerization of actin to generate small patches of the actin–myosin II cytoskeleton [50,51,52]. Since Mff functions via its affinity to membrane curvature and recruits Drp1 in mammalian cells, it is proposed that the ER tubules and actin cytoskeleton trigger a mechanical force that recruits Mff and Drp1 [53]. Interestingly, the Drp1 oligomers, Mff and Fis1, are located on the ER membrane as puncta, and these fission components are transferred to mitochondrial division site upon the ER–mitochondria contact site [54]. Therefore, the ER most likely serves as both an initiator of mitochondrial division and a platform of mitochondrial fission machinery. Similar to mitochondrial division, mitochondrial fusion also occurs at the ER-mitochondria contact site [55]. It is consistent with the fusion protein of MOM, Mfn2, which is involved in mitochondrial–ER tethering [56].

In addition to the receptor proteins and the ER-actin cytoskeleton, a mitochondria-specific polyanionic phospholipid cardiolipin also regulates mitochondrial dynamics. Cardiolipin is primarily localized at the MIM, but it is also found at the MOM [57] where Drp1 is found. Cardiolipin directly binds to an insert B region of Drp1 and promotes its oligomerization followed by stimulation of its GTPase activity, the so-called assembly-stimulated GTPase activity [58,59]. Overexpression of an insert B mutant of Drp1 with a reduced binding affinity to cardiolipin-containing membranes does not rescue the defect of mitochondrial fission in Drp1-knockout cells [60]. Cardiolipin has been also shown to interact with mitochondrial fusion proteins, such as yeast Mgm1 and human Opa1, and stimulate their GTPase activity, although these dynamin-like proteins do not contain PH domain [61,62]. Thereby, cardiolipin is important for both the fission and fusion of mitochondria.

Mutations in the mitochondrial division genes are associated with human disease [9,63]. The first reported patient was a newborn female patient with microcephaly, abnormal brain development, optic atrophy and hypoplasia, persistent lactic acidemia, and a mildly elevated plasma concentration of very-long-chain fatty acids. The patient had a dominant negative heterozygous mutation at G395D in the *DRP1* gene, manifested as a severe fission defect of mitochondria and died one month after birth [64]. Patients with mutations in the *Mff* gene are associated with early-onset Leigh-like basal ganglia disease [65]. In cells derived from these patients, fission defect in the mitochondria and impaired DRP1 recruitment are observed. Several clinical diseases have also been shown to be associated with mutations in mitochondrial fusion genes. *Mfn2* is mutated in patients with Charcot–Marie–Tooth type 2A [66], while mutations of the *OPA1* gene are associated with dominant optic atrophy [67,68].

### 2.2. In C. merolae

Unlike yeast and mammalian cells which contain numerous mitochondria and peroxisomes, the unicellular red algae *C. merolae* contains a single mitochondrion and peroxisome per cell [19]. The division of these organelles is highly synchronized by the cycles of light/dark stimulation [20,69], allowing for both snapshots of sequential events of individual mitochondrial and peroxisomal division and the visualization of the entire picture of the division machinery. Furthermore, during synchronization, division machineries can be isolated in bulk; this allows the components of the division machinery to be identified by mass spectrometry and 100% sequenced for their genomic information [70,71]. In *C. merolae*, mitochondria only divide and do not fuse, unlike the mitochondria in yeast and mammalian cells. Consistent with this membrane remodeling, *C. merolae* does not contain any mitochondrial fusion genes, such as *Mfn* and *OPA-1* [70]. The division machinery of the mitochondrion, called mitochondrion-dividing (MD) machinery, consists of three types of ring-shaped structures: the mitochondrion-dividing (MD) ring, the dynamin ring, and the FtsZ ring [23] (Figure 1B). Using transmission electron microscopy, the MD ring was identified as an electron-dense ring-like structure wrapped around the neck of dividing mitochondria [18]. There are two types of MD rings: an outer MD ring, which forms at the cytoplasmic side of the MOM, and an inner MD ring, which forms within the matrix beneath the MIM. The outer MD ring is composed of a bundle of polyglucan nanofilaments (~5 nm in width) and glycosyltransferase MITOCHONDRION-DIVIDING RING1 (MDR1) that regulates the synthesis of the filaments [72]. Molecular details of the inner MD ring are not yet clear. The dynamin ring is composed of Dnm1 and formed around the MD ring [73]. In the cytoplasm, Dnm1-positive signals are observed as 10–20 cytoplasmic puncta (dynamin patches), and Dnm1 is likely to be recruited from the dynamin patches to the mitochondrial division site [73]. The dynamin patches do not contain GTP; thus, Dnm1 is probably in a GTP-unbound form before recruitment [74]. The Dnm1 receptor proteins encoded in *C. merolae* are mitochondrial division apparatus 1 (Mda1) and Fis1 [70,71]. Mda1 is a WD40 repeat protein homologous to yeast Mdv1 and Caf4. Mda1 localizes to the mitochondrial division site prior to Dnm1 recruitment and its stable homo-oligomer is a core structure of the MD machinery [75]. Fis1 is assumed to be an adaptor between Dnm1 and Mda1, but its function in *C. merolae* remains unclear. The nucleoside diphosphate kinase (NDPK) protein DYNAMO1 was recently identified as an interacting partner of Dnm1 [25]. NDPK domain catalyzes the GTP generating reaction by transferring γ-phosphate from ATP [76]. DYNAMO1 is essential for the recruitment of Dnm1 and the constriction of Dnm1-dependent mitochondrial division by facilitating G-domain activity (both GTP binding and hydrolysis) of Dnm1 and providing GTP to Dnm1, respectively. The FtsZ ring is composed of an alphaproteobacterial-type filamenting temperature sensitive mutant Z1 (FtsZ1), a remnant of bacterial cell division apparatus [77,78]. FtsZ ring formation depends on the interaction between FtsZ1 and a bacterial ZapA-like protein, ZED [79]. An exact role of FtsZ ring in mitochondrial division is not fully understood, but is thought to play an important role in the constriction of MIM and the positioning of the MD machinery [73].

The molecular mechanisms of the initiation of mitochondrial division are not yet well understood in *C. merolae*. In yeast and mammals, the ER and actin–myosin cytoskeleton are involved in the initiation of mitochondrial division, as discussed in the previous section. In *C. merolae*, the ER extends towards the mitochondrial division site [80]. However, since *C. merolae* lacks an actin–myosin system [70,71,81], it remains unclear whether the ER is involved in the initiation of mitochondrial division. Instead of the ER and actin–myosin-mediated initiation of the mitochondrial division, the FtsZ ring is thought to play a pivotal role in the positioning of the MD machinery [73]. Light/dark cycle-induced synchronization captures the sequential event of mitochondrial division and demonstrates that the FtsZ ring forms on the matrix side of MIM followed by the recruitment of Mda1 and Dnm1 at the same site on MOM [73,75]. The exact molecular mechanism of the FtsZ ring function is not yet understood, particularly how the FtsZ ring engages in the crosstalk with the division proteins over the MIM and MOM, and it is not known what signal induces FtsZ ring formation. Understanding of these issues would be an exciting topic for the investigation in future studies.

## 3. Peroxisomal Dynamics

### 3.1. In Yeast and Mammals

**Figure 2:**
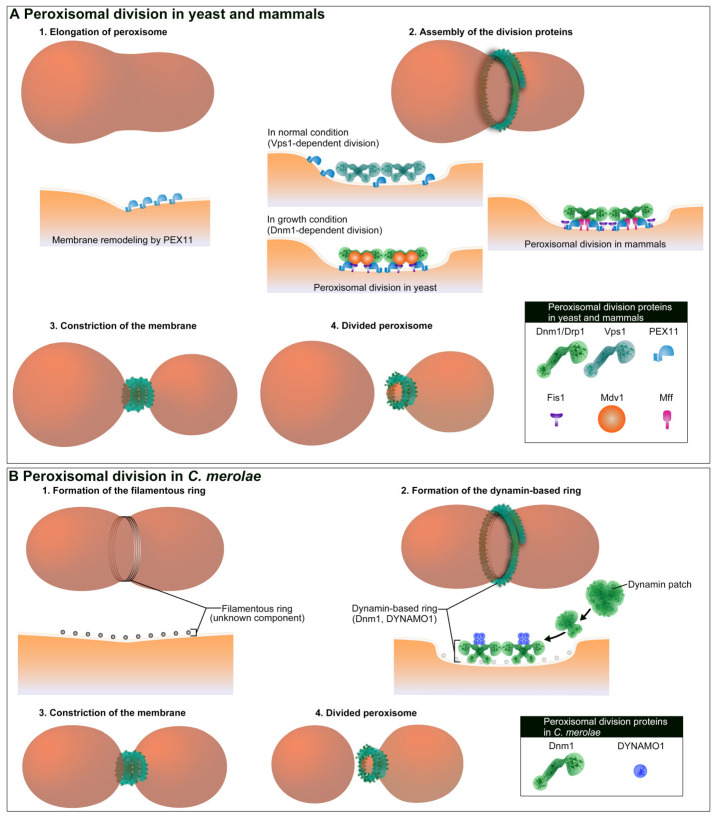
Peroxisomes are divided by Dnm1/Drp1-based membrane fission machinery. (**A**) Peroxisomal division in yeast and mammals are initiated by PEX11. In yeast *S. cerevisiae*, Vps1 and PEX11 are involved in the peroxisomal division in normal conditions. In growth condition, Dnm1, PEX11, Fis1, and Mdv1 are involved in the division. In mammals, Drp1, PEX11β, Fis1, and Mff are involved in the division. (**B**) In *C. merolae*, a peroxisome is divided by peroxisomal-dividing (POD) machinery (filamentous ring and dynamin-based ring). The filamentous ring is thought to be formed first at the division site followed by formation of the dynamin-based ring. Dnm1 is likely recruited from cytosolic dynamin patches. DYNAMO1 regulates both recruitment of Dnm1 and GTP-dependent constriction of dynamin-based ring.

Peroxisomal proliferation by “growth and division” is a widely accepted dogma in yeast and mammals [82,83]. This process involves the synthesis of peroxisomal proteins on cytosolic free-polyribosomes and their post-translational transportation into the peroxisome matrix and membrane, followed by peroxisomal division. Peroxisomal membrane protein 11 (PEX11) is the first protein to be identified that plays an important role in peroxisomal division (Figure 2A). PEX11 was originally identified as Pmp27p using yeast mutagenesis [84]. The disruption of this gene in yeast caused a significant reduction in peroxisome abundance, while its overexpression yielded the opposite result [84,85,86]. In mammals, the PEX11 isoform, PEX11β, mediates membrane growth by remodeling, deforming, and elongating the peroxisomal membrane prior to fission [87,88]. In addition to its membrane-shaping function, PEX11β is also involved in the recruitment of division factors. Peroxisome division in mammals is regulated by Drp1 [7,89], Fis1 [8,90], and Mff [38,91]. Pex11β interacts with Mff in a Drp1-dependent manner, suggesting that Mff plays a key role in the fission of the peroxisomal membrane in a concerted manner with Pex11β and Drp1 [91]. A functional complex comprising Pex11β, Mff, and Drp1 promotes the Mff-mediated fission during peroxisomal division [91,92]. The involvement of PEX11 in the regulation of peroxisomal number is conserved between yeast and mammals, although the manner of Dnm1/Drp1 action is different in yeast. In the yeast *Saccharomyces cerevisiae*, dynamin-like protein vacuolar protein sorting 1 (Vps1) is involved in peroxisomal division [93], while Dnm1, Fis1, and Mdv1 are required for division when cells are under the peroxisomal growth condition in the presence of oleate [94]. On the other hand, a study using the yeast *Hansenula polymorpha* showed that Dnm1, but not Vps1, plays a crucial role in peroxisomal division [95]. In *H*. *polymorpha*, PEX11 directly binds to Dnm1 and functions as a GTPase-activating protein (GAP) [96]. Therefore, Dnm1 function may be dispensable in a subset of cell types or environment in yeast.

As described in the previous section, the ER plays an important role in the initiation of mitochondrial division and the recruitment of Dnm1/Drp1, and also plays a pivotal role in peroxisomal proliferation. In yeast and mammalian cells, peroxisomal membrane peroxins 3 and 16 (Pex3 and Pex16) have been observed emerging from ER [97,98,99,100,101,102]. Thereby, Pex-containing pre-peroxisomal vesicle is proposed to play an important role in the biogenesis of peroxisomes followed by Dnm1/Drp1-mediated membrane fission [103]. The mechanism by which Pex-containing pre-peroxisomal vesicles participate in peroxisomal biogenesis is not yet clearly understood. However, it has been proposed that the vesicles fuse with each other or with pre-existing peroxisome to generate a larger matured peroxisome [104,105]. A recent study suggested that Pex3 is targeted to mitochondria, and Pex3-containing vesicles bud off from MOM [106]. This study also suggested that Pex3-containing vesicles fuse to Pex16-containing vesicles from the ER for peroxisome assembly. Thus, mitochondria may also be involved in the proliferation of peroxisomes, in addition to the ER. The identification of other factors involved in the regulation of the fusion of Pex-containing vesicles is another interesting topic for future studies.

As for mitochondrial division proteins, mutations in peroxisomal division proteins are also responsible for human diseases. The first reported mutation was a Chinese hamster ovary (CHO) cell mutant, ZP121, which was impaired in DRP1 with one-point temperature-sensitive and dominant negative mutation at G363D in the middle region [89]. In terms of the manifestation of peroxisomal dysmorphogenesis in humans, only three patients have been identified with a different defect in two proteins involved in the division of peroxisomes. The first patient was a severely affected female patient with a dominant negative heterozygous mutation at G395D in DLP1, who manifested a severe fission defect of both peroxisomes and mitochondria and died one month after birth [64]. The second patient with a dysfunctional DRP1 harboring a G362D mutation was more recently reported [107]. A patient with defective peroxisomal division due to a homozygous nonsense mutation in the *PEX11β* gene was reported as the 14th complementation group of the peroxisome biogenesis disorders [108,109].

### 3.2. In C. merolae

In a unicellular red algae *C. merolae*, the division of a peroxisome occurs during M phase and always takes place after mitochondrial division. The peroxisome morphology is plastic, as in mammalian cells, and is a result of the physical interaction of peroxisomes with mitochondria during mitosis [110]. Morphological changes take place in peroxisomes during their physical interaction with mitochondria, including an increase in their volume before peroxisomal division. The post-translational import of catalase is linked to an increase in the volume of a peroxisome [110]. Thus, a peroxisome in *C. merolae* proliferates via its growth and division like in yeast and mammalian cells. As such, the interaction of peroxisome with the mitochondrion may play an important role in its the morphological plasticity. The division machinery of the peroxisome, called the peroxisomal-dividing (POD) machinery, consists of two types of ring-shaped structures, namely a filamentous ring and a dynamin-based (DB) ring. Both are formed at the cytoplasmic side of peroxisomal membrane constriction site [20] (Figure 2B). The POD machinery does not contain a FtsZ ring or an electron-dense ring in the matrix side. The filamentous ring is composed of a bundle of 4-nm wide filaments, which is similar to the structure of the MD ring, but its components remain unknown. The DB ring contains Dnm1 [20] and DYNAMO1 [25], thus sharing components with the dynamin ring of the MD machinery. The DB ring is formed from a single spot on the POD machinery, called the dynamin-based ring organizing center (DOC), which functions as the nucleation site of Dnm1 [74]. GTP binding is likely to play an essential role in the assembly of Dnm1. Similar to the dynamin ring in the MD machinery, Dnm1 is recruited from the dynamin patches to the division site of the peroxisome [20,74]. DYNAMO1 provides GTP to Dnm1 on the DB ring, which is critical for generating the constriction force of the POD machinery, as well as the constriction of the MD machinery. Unlike mitochondrial division, DYNAMO1 is not involved in recruiting Dnm1 to the membrane fission site during peroxisomal division. Future studies will elucidate how *C. merolae* regulates Dnm1 recruitment during the peroxisomal division. *C. merolae* encodes *Pex11β* and thus Pex11β-Dnm1 interaction may be sufficient for the recruitment of Dnm1 to the peroxisomal division site.

## 4. Regulation of GTP during Dnm1/Drp1 Function

Dnm1/Drp1 is a core component in the division machineries of the mitochondria and peroxisomes in yeast, mammals, and *C. merolae* [11,22,23]. The key regulatory mechanisms of the Dnm1/Drp1-based division machinery include the step of recruitment of Dnm1/Drp1 to the membrane, the receptor-mediated protein recruitment, and the mobilization of GTP. We are certain that various Dnm1/Drp1 receptor proteins described in the previous sections are required for Dnm1/Drp1 recruitment. However, their regulation of GTP remains unclear. GTP is the only energy source used for Dnm1/Drp1 recruitment and membrane constriction, and GTP binding is important in the recruitment of Dnm1/Drp1. In yeast, the binding of GTP to the G-domain alters the structure of Dnm1 by exposing the insert B region involved in the membrane interaction [111]. After GTP binding, Dnm1 is able to form a highly ordered helix-shaped structure [16]. Mdv1 is known to preferentially interact with GTP-bound Dnm1 [112]. In mammals, GTP binding alters the conformation of Drp1 and allows for the interaction between Drp1 and Mid49 [113], followed by polymerization with Mff on the mitochondrial membrane [114]. Moreover, GTP and GDP influence the binding kinetics between Drp1 and actin filaments [115]. During the constriction, the Dnm1/Drp1-helical polymer is thought to demonstrate ratchet motion [17] similar to classical dynamin, which requires continuous consumption of GTP until the diameter of the membrane reaches its limit, at ~4 nm, for spontaneous membrane fission [116,117]. Although GTP binding is important for the recruitment of Dnm1/Drp1, its affinity to GTP is weak. That is, the *K_m_* of basal G-domain is at least 1 mM [118]. Dnm1/Drp1 has a relatively high rate of GTP hydrolysis on the membrane [16,118,119], at least 5000-fold higher than that of small GTPases [120]. Indeed, a study using yeast Dnm1 reported that significant constriction of lipid tubules mediated by the Dnm1-helical polymer requires extraordinarily high levels of GTP concentration (~1 mM) [17], consistent with the idea that the allosteric enhancer of G-domain is required to elevate the affinity of Dnm1 to GTP [118]. Several earlier studies have showed that Dnm1/Drp1 binding proteins, such as Mdv1 and Mff, enhance G-domain function [121,122]. In addition to these Dnm1/Drp1 binding proteins present on the MOM, cytoplasmic proteins also participate in the enhancement of the G-domain function. A study in mammalian cells found that mitochondrial fragmentation is induced by cytoplasmic cyclin C, which is released from cell nucleus in response to oxidative stress [123]. Cyclin C directly binds to Drp1 and increases the affinity of Drp1 to GTP. As GTP binding to Drp1 is required for the interaction of Drp1 with MiD49 [113], enhancing the affinity of Drp1 to GTP may play an important role in the recruitment of Drp1 to MOM from the cytoplasm. In *C. merolae*, the ortholog of Drp1, Dnm1, is likely recruited from the dynamin patches in the cytoplasm [73]. The Dnm1 in the dynamin patches is likely a GTP-unbound form [74]. The GTP-unbound form of Dnm1 is thought to be altered to GTP-bound form during the recruitment in *C. merolae*. Consistent with this, a recent study found that the NDPK protein DYNAMO1 facilitates the G-domain function and regulates the recruitment of Dnm1 to the division site of mitochondrion [25]. This indicates that GTP is needed during Drp1 recruitment and that DYNAMO1 functions as an enhancer of the G-domain function of Dnm1. The enzyme activity of NDPK is not required for the recruitment, but it is essential during the membrane constriction. During the membrane constriction in a mitochondrion and a peroxisome, DYNAMO1 localizes to the MD and POD machineries and locally generates GTP from ATP. Abolishing this activity results in stalling of the constriction of a mitochondrion and a peroxisome. Thus, DYNAMO1 is an essential GTP regulator for the Dnm1-based division machinery of a mitochondrion and a peroxisome. It is not known whether NDPK orthologs, if any, are important in Dnm1/Drp1 function in yeast and mammalian cells, while NDPK function is reported to be required during mitochondrial fusion. One of the NDPK isoforms in mammals, non-metastatic cells 4 (NME4), has been suggested to provide GTP for the OPA-1 function during MIM fusion [124,125]. Recently, another isoform NME3, was found to regulate the function of Mfn1 and Mfn2 during MOM fusion. However, in this case, GTP generation activity was not required for Mfn1 and Mfn2 functions [126]. Thus, mitochondrial fusion in mammals seems to be regulated by the NDPK protein. Fission and fusion occur in rapid succession at the same region on mitochondria [127]. Moreover, these two opposing membrane remodeling processes frequently occur at the ER–mitochondrial contact sites [49,55,128], where Drp1 and Mfn1 are colocalized [129]. Therefore, NDPK protein may also be accessible to Drp1, in addition to Mfn1. In future studies, the issue as to whether local GTP generation during mitochondrial fission is conserved in yeast and mammals will need to be addressed.

## 5. Molecular Mechanisms Underlying Local GTP Generation around the Organelle Division Machinery

Local GTP generation on dynamin family proteins has been reported in both classical dynamin and dynamin-related proteins. In *D. melanogaster*, a mutation of the NDPK gene, called *abnormal wing disc* (*Awd*), is identified as an enhancer of the *shibire* mutant phenotype, which has a defect in dynamin-dependent synaptic vesicle endocytosis [130]. In mammals, NDPK proteins are important for both clathrin-mediated and clathrin-independent endocytosis [131,132], during which the NDPK isoforms, NME1 and NME2, bind to classical dynamin and generate GTP locally on the endocytic sites. Thus, NDPK function is essential for classical dynamin. For the mitochondrial dynamics, another isoform, NME4, produces GTP on OPA-1 during MIM fusion [124,125]. For the Dnm1-dependent division of a mitochondrion and a peroxisome, the NDPK ortholog DYNAMO1 generates GTP on Dnm1 in *C. merolae* [25]. Local GTP generation by the NDPK protein is a conserved phenomenon among dynamin family proteins, although molecular mechanisms of GTP generation have a discrepancy between the two proposed models: one is that GTP is channeled within the complex of dynamin family proteins and NDPK [133,134], and the other is that GTP concentration is enriched locally around the membrane fission site [25].

**Figure 3:**
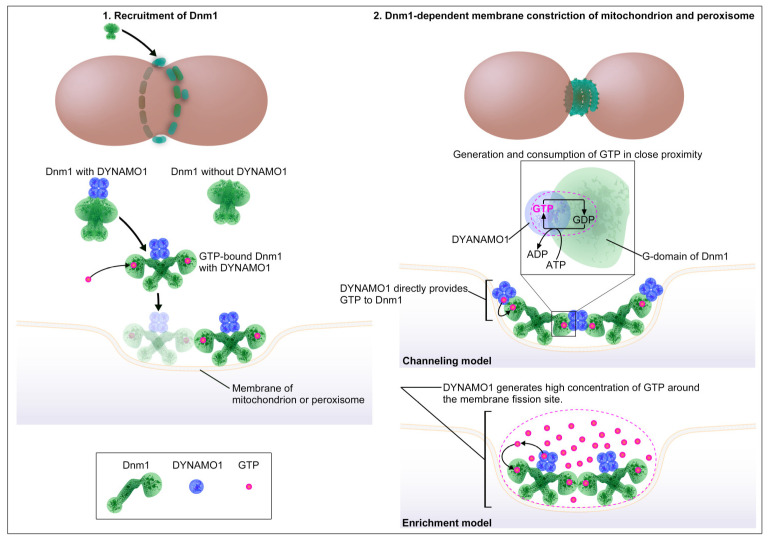
DYNAMO1 locally generates GTP for the GTPase activity of Dnm1 during the division of mitochondrion and peroxisome in *C. merolae*. During the recruitment of Dnm1 to mitochondrial membrane, DYNAMO1 binds to Dnm1 and promotes the recruitment by enhancing the G-domain function of Dnm1. During the constriction of Dnm1-based membrane fission machinery, DYNAMO1 is thought to provide GTP locally to Dnm1 by mechanisms called a channeling model or an enrichment model. In the channeling model, DYNAMO1 provides GTP in close proximity to G-domain of Dnm1. In the enrichment model, DYNAMO1 elevates local GTP concentration around the membrane fission site.

In the channeling model (Figure 3), GTP generation within the dynamin family protein-NDPK protein complex maximizes the efficiency of GTP delivery to the G-domain of Dnm1. Maximizing enzyme kinetics usually takes place in a spatial proximity within the complex of multifunctional enzymes to be separated from the diffusion equilibrium, a process known as “channeling” [135]. A well-known example of this channeling is the glycolysis reaction between glyceraldehyde-3-phosphate dehydrogenase (GAPDH) and the phosphoglycerate kinase (PGK) complex [136], and it is also proposed for the NDPK reaction [133]. The caveat of the channeling model is the unbalanced kinetics between the generation and hydrolysis of GTP. The turnover of NDPK is *K_cat_* = 600/s [137], which suggests >500–2000-fold higher enzyme kinetics than the GTPase activity of the membrane-bound classical dynamin or Dnm1 orthologs in yeast, algae, and mammals [118,119,138]. Thus, the catalytic domains of the NDPK protein and the G-domain of Dnm1 need to be in close proximity to secure a high GTP concentration ratio and to promote GTP hydrolysis without leakage of the nucleotides. However, the GTP-holding pocket in the G-domain faces each other between the adjusting helical turns in a helical polymer of Dnm1/Drp1, as well as in classical dynamin [17,116]. In this case, the interaction between the NDPK domain and the G-domain would compete with the formation of the G-domain dimer. Structural studies of the NDPK protein-dynamin family protein complex are required for further analysis and discussion of the channeling model.

The enrichment model (Figure 3) reconciles the fact that the dynamin family proteins, including Dnm1/Drp1, have a low affinity to GTP and a high rate of GTP-hydrolysis, which cannot be supported by the physiological levels of GTP. As mentioned above, the enzyme kinetics of NDPK is much higher than that of the GTPase activity of dynamin family proteins. Thus, a balance between GTP generation and consumption may not exist around NDPK-enriched membrane fission sites. In cells, NDPK forms a tetramer or a hexamer [139]. The functional membrane fission ring contains ~100 molecules of Drp1 during mitochondrial fission in mammalian cells, based on the quantification of endogenous GFP-tagged proteins [140], which are ~200 nm in diameter [53]. An accurate model for the number of G-domain dimers and the number of helix turns is required in the future studies. Given the parameters of enzyme kinetics between NDPK and Dnm1 or classical dynamin, excess of GTP may be generated around the membrane fission sites to increase the concentration of local GTP. However, the diffusion coefficient of nucleotides is ~360 µm^2^/s [141], and it is uncertain whether GTP can be locally enriched by overcoming the diffusion kinetics. This could be understood by carefully considering the coupling between the generation/consumption and diffusion of GTP. One way to locally enrich GTP is to manipulate the diffusion coefficient, as seen during the diffusion of calcium ions, whose diffusion coefficient is slowed down ~10 times as a result of molecular crowding or the restriction of movement by the cytoskeleton and cellular organelles [142,143]. Another mechanism by which local enrichment can be achieved is liquid-phase separation. Recently, the liquid-phase separation of cycling GMP-AMP synthase (cGAS) that converts GTP and ATP to cAMP is found in mammalian cells [144]. In this study, the enrichment of ATP or GTP within the cGAS-DNA liquid droplets is demonstrated. Thus, even small molecules, such as ATP and GTP, could be phase separated. However, there is only little information on the buffering elements that can slow down the diffusion coefficient of GTP, or on intrinsically disordered proteins that help in the phase separation of GTP around membrane fission sites. For example, transmission electron microscopy images of platinum replicas of an unroofed cell show that the mitochondria are surrounded with a dense cytoskeletal network in mammalian cells [52]. During clathrin-mediated endocytosis, early stage endocytic proteins have been shown to form phase separated droplets on the endocytic sites [145]. Future studies would need to examine a mobile fraction of GTP around the division sites of mitochondrion and peroxisome.

## 6. Conclusions and Perspectives

In this review, we summarize the most recent studies regarding GTP regulation during the Dnm1/Drp1-dependent membrane fission of mitochondria and peroxisomes. In addition to the importance of receptor-mediated Dnm1/Drp1 recruitment during the division of mitochondria and peroxisomes, the emerging studies have highlighted the importance of the replenishment of GTP during membrane fission. We also discussed the issues regarding the molecular mechanism underlying how local GTP generation is generated by NDPK protein on the division machineries of mitochondria and peroxisomes, as exampled by GTP channeling and GTP enrichment models. To elucidate these two models, various approaches need to be developed in future studies. To characterize the GTP channeling model, the structure of the interface between dynamin family protein and NDPK protein on the membrane needs to be elucidated. For the GTP enrichment model, the visualization of GTP concentration is vital. Recently, a circularly permutated YFP (cpYFP)-based GTP sensor was developed [146]. GTP imaging using the cpYFP could open another door in the field of NDPK protein and dynamin protein research. Currently, the working model of these proteins remains unknown. Several questions remain to be answered, such as when does local GTP generation start? How many molecules of NDPK protein are required per membrane fission machinery? What is the geometry of the NDPK proteins in the membrane fission machinery? To address these issues, the structure of the membrane fission machinery needs to be visualized using in situ cryo-electron tomography and subtomogram averaging techniques. Although these represent considerable challenges, the results will provide a new direction of research towards the membrane fission events conserved across eukaryotic cells.
